# Current Status of Older and New Purine Nucleoside Analogues in the Treatment of Lymphoproliferative Diseases

**DOI:** 10.3390/molecules14031183

**Published:** 2009-03-23

**Authors:** Tadeusz Robak, Anna Korycka, Ewa Lech-Maranda, Pawel Robak

**Affiliations:** Department of Hematology, Medical University of Lodz and Copernicus Memorial Hospital, 93-510 Lodz, Ciolkowskiego 2 Str., Poland

**Keywords:** Fludarabine, Cladribine, Pentostatin, Clofarabine, Nelarabine, Forodesine, Purine nucleoside analogues, Mechanism of action, Clinical application.

## Abstract

For the past few years more and more new cytotoxic agents active in the treatment of hematological malignancies have been synthesized and become available for either *in vitro* studies or clinical trials. Among them the class of antineoplastic drugs belonging to the purine nucleoside analogues group (PNAs) plays an important role. Three of them: pentostatin (DCF), cladribine (2-CdA) and fludarabine (FA) were approved by Food and Drug Administration (FDA) for the treatment of hematological malignancies. Recently three novel PNAs: clofarabine (CAFdA), nelarabine (ara-G) and forodesine (immucillin H, BCX-1777) have been synthesized and introduced into preclinical studies and clinical trials. These agents seem to be useful mainly for the treatment of human T-cell proliferative disorders and they are currently undergoing clinical trials in lymphoid malignancies. However, there are also several studies suggesting the role of these drugs in B-cell malignancies. This review will summarize current knowledge concerning the mechanism of action, pharmacologic properties, clinical activity and toxicity of PNAs accepted for use in clinical practice, as well as new agents available for clinical trials.

## Contents

1. Introduction
2. Pharmacology and mechanism of action
3. Clinical activity
4. Side effects and tolerability
5. Conclusions

## 1. Introduction

**Figure 1:**
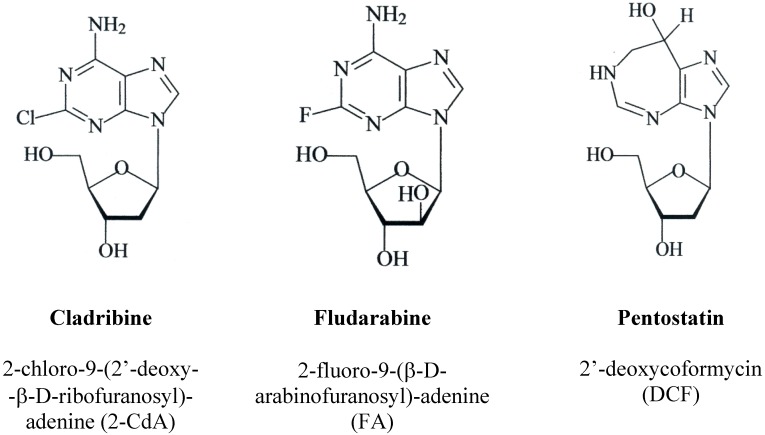
Structures of older nucleoside analogues: cladribine, fludarabine and pentostatin.

Over the past few years more and more new cytotoxic agents active in the treatment of hematological malignancies have been synthesized and become available for either *in vitro* studies or clinical trials. Among them the class of antineoplastic drugs belonging to the group of purine nucleoside analogues (PNAs) plays an important role [1,2]. In the 80-90s of the last century three of them were approved by Food and Drug Administration (FDA) for the treatment of hematological malignancies. Fludarabine (2-fluoro-9-(β-d-arabinofuranosyl)-adenine; FA), cladribine (2-chloro-9-(2’-deoxy-β-d-ribofuranosyl)adenine; 6-amino-2-chloro-9-(β-d-*erythro*-pentofuranosyl) purine, 2-chloro-2’-deoxyadenosine; 2-CdA) and pentostatin (2’-deoxycoformycin, (8*R*)-3-(2-deoxy-β-d-*erythro*-pentofuranosyl)-3,4,7,8-tetrahydroimidazo [4,5-*d*] [1,3] diazepin-8-ol, DCF) have chemical structures similar to adenosine or deoxyadenosine (Figure 1). Limited solubility of FA and difficulties in its formulation led to the synthesis of the FA prodrug – fludarabine 5’-monophosphate (FA-MP) which is commercially available.

The structure of 2-CdA differs from the naturally occurring nucleoside in the substitution of hydrogen by chlorine at the 2-position of the adenine ring. FA is a halogenated analog of arabinofuranosyladenine with fluorine at the 2-position of the adenine ring, whereas DCF is a product of fermentation cultures of *Streptomyces antibioticus* or *Aspergillus nidulans*. All three PNAs share many other similar characteristics such as uptake into the cells via nucleoside transporters, phosporylation by deoxycytidine kinase (dCK) and dephosphorylation by 5’-nucleosidase (5’-NT) [3,4]. Although all three PNAs have a similar chemical structure and act mainly by induction of apoptosis, they show different efficacy against hematological malignancies and they exhibit significant differences, especially concerning their interactions with enzymes involved in adenosine or deoxy-adenosine metabolism [5,6].

Several clinical trials have demonstrated that these drugs, used either alone or in combination with other cytotoxic agents or monoclonal antibodies, show good efficacy and acceptable toxicity profile in the treatment of indolent lymphoid malignancies [5,6]. DCF and 2-CdA are currently the drugs of choice in hairy cell leukemia (HCL) [7]. FA and 2-CdA alone or in combination with other agents are highly effective in chronic lymphocytic leukemia (CLL) and low grade B- and T-cell non-Hodgkin’s lymphomas (NHL) [8,9,10,11]. FA and 2-CdA showed also effectiveness in patients with Waldenström Macroglobulinemia (WM) and cutaneous T-cell lymphoma (CTCL) [12,13,14]. 

**Figure 2:**
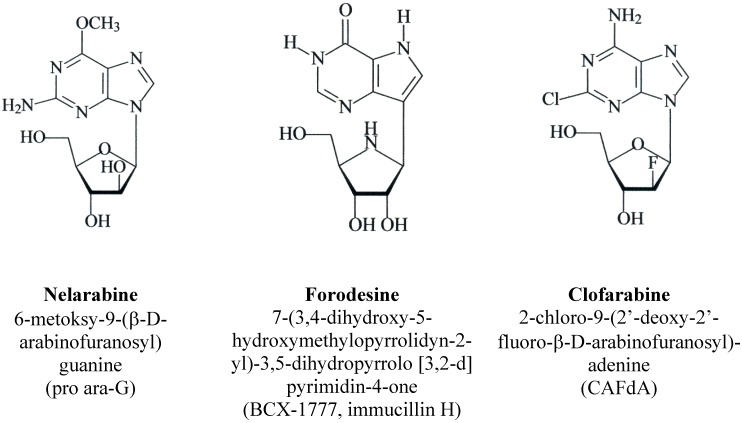
Structures of new nucleoside analogues: nelarabine, forodesine and clofarabine.

Recently three novel PNAs: clofarabine 2-chloro-9-(2’-deoxy-2’-fluoro-β-d-arabinofuranosyl) adenine; CAFdA), nelarabine [6-methoxy-9-(β-d-arabinofuranosyl) guanine; 2-amino-6-methoxy-9-(β-d-arabinofuranosyl) purine, pro ara-G] and forodesine {7-(3,4-dihydroxy-5-hydroxymethyl-pyrroli-dyn-2-yl)-3,5-dihydropyrrolo [3,2-d] pyrimidin-4-one; immucillin H, BCX-1777} have been synthesized and introduced into *in vitro* studies and clinical trials (Figure 2) [15]. CAFdA, like 2-CdA, is a deoxyadenosine analogue and exhibits efficacy in both acute myeloblastic leukemia (AML) and acute lymphoblastic leukemia (ALL), blast crisis of chronic myelogenous leukemia (CML-BP) and myelodysplastic syndrome (MDS) [16,17,18]. This agent is also active in pediatric patients with advanced leukemias. 

Nelarabine is a water-soluble prodrug of 9-β-d-arabinofuranosylguanine (ara-G), which is its active metabolite [19,20,21]. Ara-G is selectively toxic to mature T-cells and immature T-lymphoblasts as compared to B-lymphoblasts or null-cells which are resistant to ara-G. 

Forodesine belongs to a class of 9-deazanucleoside analogues which are purine nucleoside phosphorylase (PNP) inhibitors, termed immucillins [22,23]. This agent shows activity in some experimental tumors in mice. It could be useful for the treatment of human T-cell proliferative disorders and it is currently undergoing clinical trials for the treatment of T-cell NHL, which includes CTCL and T-cell ALL. However, recently forodesine seems to be useful also in the treatment of B-cell NHL and the great hopes are set on the use of the drug in B-CLL. 

This review article summarizes current knowledge about mechanism of action, pharmacokinetics, pharmacological properties, clinical activity and toxicity of older and new PNAs.

## 2. Pharmacology and Mechanism of Action

As it was mention above, the PNAs share several similar characteristics including transportation into the cells, phosphorylation to monophosphates by cytosolic dCK or mitochondrial deoxyguanosine kinase (dGK) and dephosphorylation by 5’-NT. However, there are also PNAs such as nelarabine, forodesine, pentostatin which do not need to be phosphorylated and they exhibit significant individual differences in their interaction with enzymes involved in the purine metabolism. On the other hand, all the PNAs are characterized by a similar mechanism of cytotoxicity both in proliferating and quiescent cells, such as inhibition of DNA synthesis, inhibition of DNA repair and accumulation of DNA strand breaks [5,6]. The main role in the mechanism of PNA activity plays induction of apoptosis which is the end-point of their action. This proces takes place mainly through the intristic pathway, via modulation of P53 expression or directly via binding to proteins located in mitochondrial membrane, leading to changes in mitochondrial membrane permeability. 

The first step of the cellular activity of PNA, is their uptake into the cells which occurs via at least nine different nucleoside transporter (NT) systems, which may significantly modulate intracellular drug bioavailability and responsiveness to therapy. Over the last decades two families of human NT (human equilibrative nucleside transporters; hENT and human concentrative nucleoside transporters; hCNT) have been identified and extensively studied [8,24,25]. Four of NT belonging to hENT family are sodium-independent-facilitated system and transport nucleosides down the concentration gradient . Five other hCNT are sodium – dependent system and can transport nucleosides against a concentration gradient [24]. Additionaly, ABCG2 (breast cancer resistance protein), like a first transporter directly linked to the efflux of nucleoside monophosphate analogues from mammalian cells-MRP4 (multidrug resistance protein), transport and confer resistance to PNA. These two transporeters work in parallel to affect drug cytotoxicity and tissue distribution [26]. The single – nucleotide polymorphism in human MRP4 dramatically reduces MRP4 function by impairing its cell membrane localization [27]. 

### 2.1. Fludarabine

**Figure 3:**
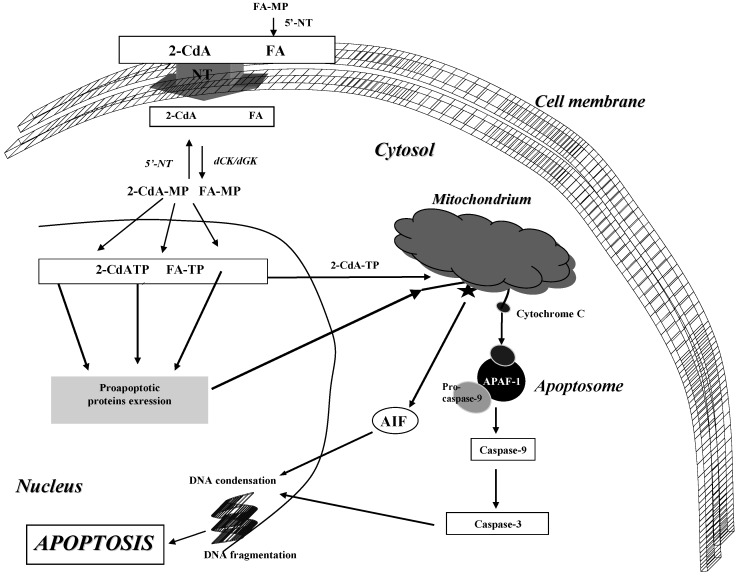
Mechanism of 2-CdA and FA action.

FA, like other PNAs, permeates the cell via different NT systems. However, NT accept only dephosphorylated forms, so prior to entering the cell, commercially available FA-MP is rapidly dephosphorylated in plasma to FA by ecto-5’-NT. FA can be transported into the cells via such NT as hENT1, hENT2, hCNT2, hCNT3 (Figure 3) [24,28,29,30]. After the uptake into the cells, FA must be converted by dCK or dGK into its triphosphate form, which is the active metabolite required for its cytotoxicity [31]. At the same time the phosphorylated metabolite is dephosphotylated by 5’-NT. In lymphoid cells, a high ratio of dCK/5’-NT activity favors the accumulation of phosphorylated metabolites which inhibit various processes involved in DNA and RNA synthesis, modulate apoptosis, influence cell-cycle control or signal transduction pathways [32]. 

2-CdA-2-chloro-2’-deoxyadenosine; FA-MP-fludarabine monophosphate; FA-fludarabine; 2-CdA-MP-2-CdA monophosphate; FA-TP-fludarabine triphosphate; 2-CdA-TP-2-CdA-triphosphate; NT-nucleoside transporter; 5’-NT-5’-nucleotidase; dCK-deoxycytidine kinase; AIF-apoptosis-inducing factor; APAF-1-apoptotic protease activating factor;

FA-MP is converted to FA by an apparent first-pass metabolism within five minutes after rapid intravenous (i.v.) infusion and linear relationship between the dose of FA-MP and FA concentration was observed [33]. Peak intracellular levels of FA-TP, an active metabolite of FA were observed within 3-4 hours after termination of FA-MP infusion. Plasma concentrations of FA were fitted to a three-compartment open model. The half-life (T_1/2 _α ) was five minutes, followed by an intermediate phase (T_1/2 _β) of 1.38 hours and a prolonged terminal phase (T_1/2 _γ) of 10.41 hours. The mean apparent value of distribution (Vdr) was 62.28 ± 16.88 L/m^2 ^, and the mean steady-state volume of distribution (Vdss) was 44.17 ± 10.82 L/m^2^. Since Vdr is greater than Vdss, these data indicate extensive tissue binding of FA [34]. FA is eliminated from the body in the urine as approximately 60% of this drug is present in the urine within 24 hours of administration. Total body clearance of FA (67.98 mL/min/m^2 ^± 19.58) correlates with creatinine clearance [30]. FA-MP is usually administered at a dose of 25 mg/m^2^/day in a 30 min infusion or i.v. bolus injection for 3-5 days and the courses are repeated every 3-5 weeks [33,35]. 

An oral formulation of FA-MP has recently become commercially available. Biovailability of oral FA-MP is about 50% to 75% of the i.v. dose with a similar terminal T_1/2_, and is unaffected by food [1,36]. It has been recently reported that an oral dose of approximately 40-50 mg/m^2^ /day of FA-MP provids systemic exposure equivalent to 25 mg/m^2^/day of routinely given i.v. [33]. The oral formulation of FA-MP is easier to use and can be administered on an out-patient basis [37].

### 2.2. Cladribine

Intracellular phosphorylation of 2-CdA (2-chloro-9-(2’-deoxy-β-d-ribofuranosyl)-adenine;2-chloro-2’-deoxyadenosine) is necessary for any cytostatic effects to occur. Like FA, it is phosphorylated by dCK and accumulated as 2-chlorodeoxyadenosine triphosphate (2-CdA-TP) [38]. The nucleotide that is formed does not readily exit from the cells through the cell membrane and therefore accumulates inside the cell. This metabolite disrupts cell metabolism by incorporating into the DNA of the actively dividing cells and freezes cell cycle at S phase [38,39]. The uptake of this drug into the cells is also mediated through one or more of NT and may significantly modulate intracellular drug bioavailability and, consequently, responsiveness to therapy. The drug is transported via hENT1, hENT2, hCNT2, hCNT3 [24]. ABCG2 transports and confers resistance to cladribine and interact with this drug by a site distinct from the prazosin binding site as shown by their inability to displace the substrate analogue [26]

In contrast to other antineoplastic drugs, but similarly to other PNA, 2-CdA is cytotoxic to both proliferating and quiescent cells. Several pathways are suggested to be responsible for the mechanism of their action. The cytotoxicity of 2-CdA in the proliferating cells is mainly due to either suppression of DNA synthesis via inhibition of DNA polymerases, or incorporation into DNA, or inhibition of DNA repair and accumulation of DNA breaks. In addition, 2-CdA strongly inhibits ribonucleotide reductase (RR) activity leading to imbalance in deoxynucleotide triphosphates (dNTP) pool, and via endonuclease activation leads to DNA strand breaks [40,41]. It is also suggested that the cytotoxic mechanism of 2-CdA may originate not only from the suppression of RR and DNA polymerases, but also from the inhibition of deoxyadenosine deamination and phosphorylation, with subsequent inactivation of 5-adenosylhomocysteine hydrolase (SAHM) as a natural consequence of adenosine accumulation [42]. It is also known that RR and two other enzymes: 5’-NT and PNP participate in catalysing purine nucleoside degradation [43]. DNA breaks activate poly(ADP-ribose) polymerase (PARP), leading to nicotinamide adenine dinucleoside (NAD) and adenosine triphosphate (ATP) depletion and can kill cell along the necrosis pathway [1]. On the other hand, DNA damage induces P53 expression and eventually leads to apoptosis which is the main mechanism of 2-CdA cytotoxicity. Recently, it has been found that P53 may also contribute to cytotoxicity in dividing cells by binding to DNA with incorporated 2-CdA as *P53*/DNA-PK complex [44]. In quiescent cells 2-CdA-TP interferes with proper repair of DNA and leads to a total disruption of cellular metabolism via accumulation of breaks in DNA strand, which in turn leads to P53 expression and consequently to induction of apoptosis [8]. In these cells, however, both *P53*-dependent and *P53*-independent cytotoxicity mechanisms are suggested to occur [45,46]. 

Apoptosis induced by 2-CdA can be mediated either via DNA damage and P53 protein expression or directly via mitochondrial permeability transition pores (mtPTP) [43]. Inhibition of DNA repair and accumulation of DNA breaks leads to *P53* expression which plays a key role in the control of apoptosis and cell cycle and influences the BCL-2 protein family with antiapoptotic properties as well as BCL-2 like proteins such as BAX, BCL-xs and BAK which have proapoptotic action [47,48]. The most important however is the relationship between BAX and BCL-2. BAX activity leads to mitochondrial changes resulting in the release of cytochrome c from mitochondrium to cytosol and its binding with apoptotic protease activating factor (APAF-1) and activation of procaspase-9. APAF-1, cytochrome c and procaspase-9 form a complex termed apoptosome which after binding with 2-CdA-TP leads in consequence to activation of caspase-9 cascade (intrinsic pathway) [49,50] (Figure 3). However, the role of BAX protein in PNA-induced apoptosis is still controversial. Bosanquet *et al*. [50] demonstrated that BAX expression correlates with sensitivity to doxorubicin (DOX), cyclophosphamide (CY) and chlorambucil (Chl), but not to 2-CdA. This finding suggests that the resistance to 2-CdA in CLL cells is due to activation of pathways other than those activated by anthracyclines and alkylating agents. On the other hand, it is also known that 2-CdA-TP induces apoptosis by direct mitochondrial pathway [20]. It can directly bind to proteins located in mitochondrial membrane resulting in the release of either cytochrome and formation of apoptosome or apoptosis inducing factor (AIF). The AIF protein leads to chromatin condensation and DNA fragmentation without the caspase-9 cascade activation [50]. 

Induction of apoptosis by 2-CdA via the death receptor Fas/CD95 on extrinsic pathway remains controversial [48,49]. Nomura *et al*. [46] have shown that apoptosis of leukemic cells in response to 2-CdA could be mediated through Fas/FASL pathway. In contrast, Klopfer *et al*. [51] have documented that apoptosis induced by 2-CdA is independent of CD95/Fas receptor or of direct effect on APAF-1, and rather follows the mitochondrial signaling pathway of cytochrome and caspase-9 cascade activation.

The clinical pharmacokinetics of 2-CdA have been evaluated in patients with lymphoproliferative diseases and acute leukemias. The initial evaluation of this agent was conducted using a continuous infusion for 5 to 14 days [52]. However, in subsequent studies short i.v. infusions, oral and subcoutaneus (s.c.) administrations have been utilized [53,54,55]. Plasma concentrations of this agent were determined either with high performance liquid chromatography or radioimmunoassay [56,57].

In the treatment of lymphoid malignancies 2-CdA is usually administered i.v. in a dose of 0.12-0.14 mg/kg in continuous or 2-hour infusion. The mean plasma concentration is 2,0 mM, wherease the plasma drug level during the 24-hour infusion is 23 mM [57]. Lilemark and Juliusson [56,58] showed that the T_1/2 _γ of plasma 2-CdA was about eight hours. The same authors have shown a T_1/2 _γ of 6.3 hours after a 2-hour infusion of 2-CdA which permits the drug to be administered as an intermittent infusion without loss of antitumor activity [56]. In population pharmacokinetics study performed by Lindelman *et al* [59] clearence of 2-CdA was 39.3 L/hour with a large interindividual variability. The T_1/2_ for the terminal phase was 16 hours.

2-CdA can also be administered orally. However, oral administration of 2-CdA solution induces degradation to 2-chloroadenine, which can be overcome by doubling the dose. The bioavailability after oral administration of 2-CdA is 35-55% [60]. Maximum oral bioavailability after the dose of 0.18 mg/day dissolved in phosphate buffer solution is 55% [54]. 

The study of Juliusson *et al*. [61] has shown that 2-CdA can also be administered in s.c. injections. It was shown that there is 100% 2-CdA bioavailability after subcutaneous administration with a high peak concentrations of short duration with an area under the curve (AUC) very similar to those achieved with a 1-hour i.v. infusions [54]. After the subcutaneous dose of 3.4 mg/m^2^ body surface area, the median AUC in plasma following the first injections was 567.5 μmol/L/h (range 140 to 356 μmol/L/h). The median T_1/2 _of 2-CdA in plasma was 7.9 hours following s.c. injection, as compared to 9.9 hours following bolus infusions [54,61]. It should be noted that there were no local or other side effects using the s.c. route, except for the systemic effects. 

### 2.3. Pentostatin

DCF (2’-deoxycoformycin) is isolated from fermentation cultures of *Streptomyces antibioticus* or *Aspergillus nidulans*. Chemically the drug is a tetrahydroimidazo{4,5-d [1,3]diazepine ring linked to deoxyribose (Figure 1). DCF is an irreversible inhibitor of adenosine deaminase (ADA), which results in intensive conversion of adenosine (Ado) and deoxyadenosine (dAdo) into inosine nucleoside derivatives [62]. ADA is a critical enzyme in the salvage of purines, catalyzing the irreversible deamination of adenosine to inosine and deoxyadenosine to deoxyinosine [63]. ADA prevents the accumulation of deoxyadenosine-5’-triphosphate (dATP), which becomes cytotoxic at high concentrations [9]. In consequence, DCF-mediated inhibition of ADA causes accumulation of triphosphate metabolites in cytosol and inhibits DNA synthesis in dividing cells. Induction of DNA breaks and inhibition of DNA repair as well as inhibition of RNA transcription play an important role in the mechanism of DCF action [62,64]. Moreover, increased levels of Ado or dAdo derivatives in non-dividing cells can lead to unbalanced ratio between *S*-adenylmethionine and *S*-adenyl-homocysteine, thus impairing the synthesis of methylated nucleosides [62,64]. Inhibition of RNA transcription, induction of DNA breaks and inhibition of DNA repair play the important role in the mechanism of DCF action.

Studies on pharmacokinetics of DCF revealed that plasma concentration measured at one hour after i.v. administration was proportional to the dose of pentostatin in the range 7.5 to 30 mg/m^2^ [64]. Plasma concentration of DCF was best fitted to a two-compartment open model [65]. The mean T_1/2 _α ranged from 8.72 to 60 minutes, followed by the terminal phase of 4.93 to 10 hours [66,67]. The mean apparent Vdr was 23.1 ± 6.16 L/m^2^, and the mean Vdss was 20.1 ± 5.01 L/m^2^. The similarity of these values suggests that DCF does not achieve extensive tissue binding [65]. The drug crosses the blood-brain barrier with concentration in the CSF of 10 to 20% of that in plasma [68]. Pentostatin is predominantly excreted unchanged by kidneys and approximately 95.9% of the administered dose is present in the urine. Total body clearance is 52.4 ± 16 mL/min/m^2^ and correlates with creatinine clearance [65]. In the treatment of lymphoid malignancies, DCF is generally administered at a dose of 4 mg/m^2^ as an i.v. bolus or short (20 to 30 min) infusion [69].

### 2.4. Clofarabine

Clofarabine [2-chloro-9-(2’-deoxy-2’-fluoro-β-d-arabinofuranosyl) adenine] belongs to the second generation of PNAs. It has been synthesized to combine the most favorable pharmacokinetic properties of fludarabine and cladribine [70,71,72]. The structure of CAFdA, similarly to 2-CdA, consists of deoxyadenosine with substitution of hydrogen by chlorine at the 2-position of the adenine ring causes electronic changes that make the amino group resistant to deamination by ADA (Figure 2) [32]. Substitution of a fluorine atom at the arabinosyl configuration at the 2’-position of the carbohydrate decreased the susceptibility of CAFdA to phosphorolytic cleavage by PNP. Additionally, it makes CAFdA more acid stable and leads to an increase of its oral bioavailability [73].

Clofarabine is a slightly lipophilic drug entering into the cells also via NT systems, and at higher concentration and longer exposure time, by passive diffusion across lipid membranes [74]. The drug is transported via hENT1, hENT2, hCNT2 and hCNT3 (24). Like other PNA, CAFdA, after the uptake into the cells, is converted by dCK to the 5’-monophosphate metabolite, and then by mono- and diphosphokinases to the active 5’-triphosphate form (CAFdA-TP). dCK phosphorylates CAFdA with a 50% lower Michaelis-Menten constant (K_M_) but a 4-fold higher maximum velocity (V_max_) than the physiological substrate deoxycytidine (dCy) [75,76]. Additionally, clofarabine was reported to be more efficient substrate for purified recombinant dCK than FA and 2-CdA [75,77]. Since CAFdA inhibits RR, it has been suggested that this drug may increase cellular dCK activity and enhance its own activation [71]. dCK is known to be rate-limiting enzyme for many of adenosine analogues, but is not rate limiting with clofarabine and clofarabine 5’-monophosphate that serves as a reservoir for the formation of 5’-triphosphate metabolites [78].

Clofarabine acts via three mechanisms of action: incorporation into DNA, inhibition of RR, and induction of apoptosis. The clofarabine triphosphate form (CAFdA-TP) is required for its cytotoxicity. CAFdA, in contrast to other anticancer drugs but similarly to the whole PNA class, is active both in mitotic and quiescent cell cycle phase [71]. The cytotoxicity of CAFdA-TP in the dividing cells is mainly due to the inhibition of either DNA polymerases or RR, leading to disequilibrium in deoxynucleotide triphosphates pool and via endonuclease activation results in DNA strand breaks [79]. The incorporation of the monophosphate of CAFdA into DNA by DNA polymerase α results in chain termination and strand breakage. The inhibition of RR in consequence reduces the amount of intracellular dNTP available for DNA synthesis, mainly deoxycytidine triphosphate (dCTP) and dATP but not deoxytymidine triphosphate (TTP) [79]. The depletion of the dCTP pool is sufficient to limit DNA synthesis, and reduction in dATP concentration allows CAFdA-TP to compete with dATP for incorporation into DNA [71,79]. In comparison with FA and 2-CdA, CAFdA inhibits more completely both RR and DNA polymerases [75,77]

**Figure 4:**
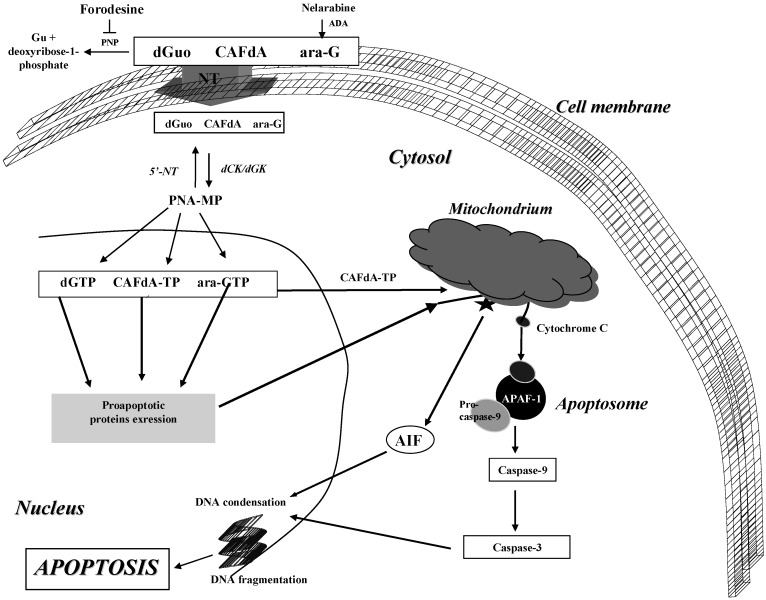
Mechanism of clofarabine, nelarabine and forodesine action.

Clofarabine can also induce apoptosis, and both *P53*-dependent and *P53*-independent mechanisms of cytotoxicity are suggested [6]. Additionally, CAFdA-TP similarly to 2-CdA can induce apoptosis by binding directly to proteins located in mitochondrial membrane. Thus, the direct mitochondrial effects of CAFdA and 2-CdA may explain why these drugs are toxic to CLL cells at concentrations 5-10-fold lower than FA (Figure 4) [72,80].

CAFdA-clofarabine; PNA-MP-purine nucleoside analogue’s monophosphate; CAFdA-TP-clofarabine triphosphate; Gu-guanine; dGuo-deoxyguanosine; ara-G-arabinosylguanine; ara-GTP-arabinosyl triphosphate; dGTP-deoxyguanosine triphosphate; ADA-adenine deaminase; NT-nucleoside transporter; 5’-NT-5’-nucleotidase; dCK-deoxycytidine kinase; AIF-apoptosis-inducing factor; APAF-1- apoptotic protease activating factor

The pharmacokinetics of CAFdA were evaluated both in adult and pediatric patients in the phase I studies [78,81,82]. During the initial phase I clinical studies conducted in patients with hematological malignancies and refractory solid tumours the maximum tolerated doses (MTD) and the dose-limiting toxicities (DLT) were assessed [78,82,83]. CAFdA was given as a 1-hour infusion daily for five days, every 3 to 6 weeks. The recommended dose for phase II studies was 40 mg/m^2^ i.v. for five days in patients with acute leukemias, MDS and CML-BP [78,81]. In patients with chronic lymphoproliferative disorders (LPDs) and solid tumors, the DLT was myelosuppression at the dose of 15 mg/m^2^, therefore in these patients CAFdA at the doses of 2 mg/m^2^ or 4 mg/m^2^ i.v. daily for five days were recommended for phase II clinical trials [82].

After administration of CAFdA at a dose of 40 mg/m^2 ^as a 1-hour i.v. infusion, the median plasma level of CAFdA was 1.0 μM (range 0.26-1.94μM) [81]. The peak level of CAFdA in plasma occurred at the end of the infusion and a linear increase in plasma CAFdA concentration with increasing doses was observed [78]. Clofarabine was only 47% bound to plasma proteins, predominantly to albumin and showed extensive tissue distribution, having a V_dss_ of 179 L for a 40-kg patient (about 4.5 L/kg) [71,78]. CAFdA appeared to be eliminated in a biphasic manner with faster kinetics during the first 6 hours followed by slower kinetics up to 24 hours [71,82,83]. The pathway of elimination is primarily via renal excretion, and the renal clearance was 10.8 L/h/m^2^ with 57% of the dose excreted unchanged in the urine [71,78]. The total systemic clearance for clofarabine was 28.8 L/h/m^2^. 

Similar to plasma CAFdA levels, the concentration of intracellular CAFdA-TP measured in circulating leukemic blasts varied among patients and the median value was 15 μM with a range of less than 1 μM to 44 μM [81]. No differences were observed between myeloid and lymphoid leukemic blasts regarding the retention of intracellular CAFdA-TP [81]. 

### 2.5. Nalarabine

Nelarabine [6-methoxy-9-(β-d-arabinofuranosyl) guanine] is a prodrug of 9-β-d-arabinofuranosyl-guanine (ara-G). It belongs to the guanosine analogues in which at the 6-position of guanine ring the hydrogen of a hydroxide group is substituted by a methoxy group (Figure 2) [84]. 

In blood nelarabine is rapidly demethoxylated to ara-G by ADA and afterwards it permeates the cell via *p*-nitrobenzylthioinosine-sensitive and insensitive hENTs [85]. In contrast to other PNA, the active metabolite of nelarabine is not its triphosphate form but instead ara-G is phosphorylated by either dCK to cytosolic ara-GTP or dGK to mitochondral ara-GTP [86]. On the other hand, deactivation of nucleosides occurs partly through either deamination by ADA or dephosphorylation by 5’-NT and partly by cleavage of glucosidic bond by the active bacterial enzyme PNP [86].

The first limiting step in the formation of ara-GTP is initial phosphorylation of ara-G to monophosphate (ara-GMP) and afterwards via diphosphate (ara-GDP) to its triphosphate form (ara-GTP), which upon incorporation into DNA, results in DNA breaks and leads to apoptosis [21]. The ara-G competes with native deoxynucleosides as a substrate for incorporation into DNA by DNA polymerases. It results in inhibition of DNA synthesis and initiation of apoptosis. On the other hand, ara-G is also able to induce apoptosis in T cells by involvement of Fas/FasL system resulting in release of soluble FasL (sFasL) and triggering death receptor mediating cell death in the bystander cells. In contrast, myeloid and B-cells accumulate lower levels of ara-GTP and arrest in S-phase, blocking any apoptotic signaling [87]. Nelarabine has consistently been reported to be more cytotoxic in T-lineage than in B-lineage leukemias [21,88,89]. Beeslay *et al*. [90] have confirmed above results and shown that nelarabine is particularly effective in T-cell ALL, however a subset of patients with B-lineage ALL might also be sensitive. 

Pharmacokinetics of nelaribine was evaluated in patients with hematologic malignancies. Nelarabine levels reached a median peak value of 18 μmol/L at the end of the infusion. After the end of the nelarabine infusion, the concentration of this agent declined with time in a monoexponential fashion with a mean T_1/2_ of 14.1 minutes in pediatric and 16.5 minutes in adult patients [91]. The maximum plasma concentration (C_max _) of ara-G occurred within 15 minutes after the end of the 1-hour infusion of nelarabine. The C_max _of ara-G ranged from 11.6 μmol/L to 308.7 μmol/L at nelarabine doses of 5 to 75 mg/kg and was linearly related to these doses. In contrast to nelarabine, ara-G was eliminated from plasma slowly with a median T_1/2_ of 4.2 hours (range 1.8 to 5.6 hours) [91]. The pharmacokinetic parameter estimates of ara-G have showed that blasts from patients with T-ALL or T-lymphoid CML-BP accumulated relatively greater levels of ara-GTP compared to other lineages [92]. It was observed that clearance (CL) of ara-G was higher in pediatric patients as compared to adults, although it may be associated with age-related differences in renal function. 

### 2.6. Forodesine

Forodesine belongs to 9-deazanucleoside analogues which are termed immucillins. The structure of these agents based on ”nitrogen in the ring” of d-ribofuranose and C-glycosidic bond analogues of natural nucleosides [93,94]. These agents are C-nucleosides containing nitrogen atom in the ring of d-ribofuranose. Forodesine, chemically described as [7-(3,4-dihydroxy-5-hydroxymethylpyrrolidyn-2-yl)-3,5-dihydropyrrolo-[3,2-d] pyrimidin-4-one], is one of the most potent members of this group (Figure 2) [93,94,95]. In contrast to other PNAs, for which the active forms are nucleoside triphosphates, in case of forodesine, the nucleoside is the active form. The drug does not act via incorporation into DNA and inhibition of DNA synthesis, but displays a highly selective PNP inhibitory action [22]. 

In the mechanism of forodesine action, apart from PNP two other enzymes, dCK and dGK, are involved. Normally the affinity of dGuo is higher for PNP than for dCK. Nevertheless, in the absence of PNP plasma dGuo reaches high level and is not cleaved to Guo, but instead of it is intracellularly converted mainly by dCK or dGK to dGMP, and then by mono- and diphosphokinases to the active dGTP [15,96]. 

However, similarly to other purine nucleoside, the first step of the cellular activity of dGuo is their uptake into the cells which occurs via one or more of NT. In order to be biologically active, dGuo needs to be phosphorylated intracellularly, and its cytotoxicity depends on accumulation of dGMP in the cells. Accumulation of dGTP as a result of forodesine action leads to RR inhibition, imbalance of dNTP pool, inhibition of either DNA synthesis or DNA repair, which results in accumulation of DNA breaks [97,98]. DNA damage due to inhibition of DNA repair and accumulation of DNA breaks leads to the P53 expression which plays a key role in the control of apoptosis and cell cycle [6,15]. It has been recently demonstrated that post-translational modification of P53 is required for its stability and activation following cell stress and is a determinant of P53-mediated apoptosis [99]. Balakrishnan *et al*. [22] have recently shown that forodesine treatment also results in post-translational modification of P53, its stability, and P53 dependent P21 activation. 

On the other hand, similarly to other cells which were treated with PNAs, in cells showing an accumulation of dGTP as a result of forodesine action, apart from intrinsic cell death pathways connected with DNA damage and P53 protein expression, a direct mechanism connected with MPT pore and resulting in the release of proapoptotic proteins has been also described (Figure 4). Additionally, an extrinsic pathway via death receptor Fas/CD95 can be also important [5,22].

The bioavailability after oral administration of forodesine in mice was 63%. At a single dose of 10 mg/kg the drug increased dGuo accumulation to approximately 5 μM [17]. After increasing the oral administration of forodesine up to 10mg/kg no further increase in dGuo was observed. *In vivo* studies in primates revealed that oral and i.v. administration of forodesine induced a rapid elevation of plasma 2’-dGuo and that oral dosing at 8.8 and 17.6 mg/kg were at least equivalent to 4.4 mg/kg i.v. twice daily in effecting 2’-dGuo accumulation. Increasing the i.v. dose of the drug did not increase dGuo accumulation; however plasma dGuo concentration remained elevated longer. In contrast, the increase of oral doses resulted in elevated plasma dGuo accumulation [100]. In human blood dGuo is degraded rapidly with a T_1/2_ of 12 sek. and for achieving dGuo concentration needed to a significant reduction of T-cell function the continuous inhibition of PNP greater than 95% is required [96,98].

## 3. Clinical activity

The activity of PNAs in the treatment of lymphoid malignancies is documented in several clinical trials. 2-CdA and DCF are useful agents in the treatment of HCL. These drugs are also active in the treatment of low -grade B- and T-cell NHL. Moreover, FA, 2-CdA and DCF administered in CLL patients as monotherapy as well as a combined treatment either with other cytotoxic agents or monoclonal antibodies demonstrate high response rates. Two of new PNAs – forodesine and nelarabine – are also active in lymphoid malignancies, especially in acute T-cell lymphoblastic leukemia.

### 3.1. Hairy cell leukemia

2-CdA and DCF are the drugs of choice in the treatment of HCL. In contrast, FA is less active in the treatment of this disease and only minor clinical experience has been reported so far [1,2]. Piro *et al*. [101] were the first to describe sustained complete response (CR) in HCL patients who had undergone splenectomy and received a single continuous i.v. infusion of 2-CdA for seven days. Further multiple studies on larger groups of patients demonstrated that 2-CdA induces durable and unmaintained CR in about 80% of patients after a single course of therapy [105,106,107,108,109,110,111,112,113,114,115,116,117,118,119,120]. 2-CdA is administered either as a continuous i.v. infusion at a dose of 0.09 mg/kg over a 5-7 days period or as a 2-hour i.v. infusion at a dose of 0.12 mg/kg for 5-7 days [55,102,103]. However, similar results were achieved when the drug was given in s.c. injection [104]. Preliminary observations indicated that a CR following 2-CdA administration was durable even without maintenance therapy, so this drug was considered as potentially curative against HCL [105,106,107,108,109]. However, a long-term clinical follow-up of patients who had entered CR also after a single course of 2-CdA revealed that about 20% of them relapsed [109,110,111]. 

2-CdA is highly effective in relapsed disease. Goodman *et al*. [109] has reported the results of re-treatment with this agent. The overall response rate of relapsed patients who were retreated with 2-CdA was 90% with 75% achieving a CR and 10 (17%) a partial response (PR). The median second response duration for all responders was 35 months while the median time to first relapse was 42 months. The second relapse was observed in 20 patients and 10 of them received second re-treatment with 2-CdA. Overall response rate was 80% including 60% of CRs. The median of the third response duration was 20 months.

2-CdA is also an effective drug when administered at a dose of 0.15 mg/kg in a 2-hour infusion once a week over 6 courses. In the study by Lauria *et al*. [112] 22/30 (73%) patients with HCL achieved a CR and 8 (27%) patients achieved a PR when 2-CdA was given in this mode. This type of drug administration may be less toxic and reduces the risk of infection complications in comparison with standard 2-CdA daily regimens. In our randomized study we compared weekly administration of 2-CdA (0.12 mg/kg in a 2-h i.v. infusion once a week for 6 weeks) with daily administration (0.12 mg/kg in a 2-h i.v. infusion for 5 consecutive days) [113]. The updated results of this study indicate that both CR and overall response (OR) rates were similar in compared groups. There was no statistically significant difference in toxicity between groups, except for thrombocytopenia. It seems, however, that daily administration of 2-CdA may more frequently induce thrombocytopenia and neutropenia and lead to more frequent infections. 

In the study of Juliusson *et al*. [114,115], 73 patients were given 2-CdA as a subcutaneous injection once daily for seven days. Fifty-nine patients (81%) achieved a durable CR after one (n = 55) or two courses and 10 had a PR. With a median follow-up duration of 20 months no patients had a clinical relapse. Similar study was performed by von Rohr *et al*. [104]. In this multicenter phase II trial, 62 patients received a first cycle with 2-CdA at a dose of 0.14 mg/kg/day by s.c. bolus injection for five consecutive days. Complete and partial response were seen in 47 (76%) and 13 (21%) patients, respectively. At a median follow-up of 3.8 years, progression after PR was seen in seven patients and relapse after CR was seen in eight patients.The above two trials may indicate that 2-CdA given by s.c. bolus injections is very effective in HCL and more convenient for patients than continuous i.v. infusion. 

In 1984, Spiers *et al*. [116] first demonstrated that DCF is a highly active agent in HCL. Subsequently the Eastern Cooperative Oncology Group conducted a multicenter study of DCF in patients with this disease [117].The initial dose was 5 mg/m^2^ administered on three consecutive days every four weeks, but this was later modified to 5 mg/m^2^ given on two consecutive days every two weeks. Among the 27 eligible patients, the response rate was 96% with 16 (59%) patients who entering a CR and 10 (37%) patients achieving a PR. These observations were later confirmed by several centers [118,119,120,121,122,123,124,125,126,127,128,129]. In these studies DCF was usually used at a dose of 2-4 mg/m^2^ i.v. every second week although other schemes were also applied. Ranges of CR rate varied from 40 to 90% regardless of prior treatment with IFN-α or splenectomy [120,121,122,123,124,125,126,127]. The drug is well tolerated in HCL, especially when neutropenia is not severe or there is no history of life-threatening infections. Myelosuppression is the main toxicity and may require delays in planned chemotherapy schedule [119].

A randomized study comparing DCF with IFN-α has demonstrated that DCF produced a higher CR and PR rate with more durable responses in HCL [129]. In that study patients were randomized to receive either IFN-α (3x10^6^U s.c. three times per week) or DCF (4 mg/m^2^ i.v. every two weeks). Patients who did not respond to initial treatment were crossed over. Among IFN-α patients, 17 of 159 (11%) achieved a confirmed CR or PR. Among DCF patients, 117 of 154 (76%) achieved a confirmed CR and 121 of 154 (78%) had a unconfirmed CR (CRu) or PR. Response rates were significantly higher (p < 0.0001) and relapse-free survival was significantly longer with DCF than IFN-α (p < 0.0001). Subsequently, long-term data on duration of overall survival and relapse-free survival data from this study were reported. Estimated a 5- and 10-year survival rates for all patients were 90% and 81%, respectively. Moreover, only two of 40 deaths were attributed to HCL. Other long-term observations also confirmed that DCF induces a high rate of long-lasting CR both in patients previously untreated and in patients pretreated with IFN-α or splenectomy [120,121,122,123,124,125,126,127]. 

2-CdA and DCF seem to induce similar high response rates but only a large randomized trial with the two agents will be able to evaluate the CR rates, duration of response, recurrence rates and adverse events, which have appeared to be comparable so far. Recently, Else *et al*. [129] have reviewed a series of 219 patients with HCL, with a median follow-up from the diagnosis of 12.5 years (range 1.0 - 34.6 yrs) treated either with DCF (n = 185) or 2-CdA (n = 34), to compare the effectiveness of these agents. Overall response to DCF was 96% with a CR in 81% and median disease free survival (DFS) of 15 years. Response to first line 2-CdA was 100% with a CR in 82% and DFS of 11+ years. Disease-free survival (DFS) showed no plateau in both groups. The relapse rates at five years and ten years were 24% and 42%, respectively, with DCF and 33% and 48% with 2-CdA. Survival at ten years was 96% in DCF group and 100% in the 2-CdA group. This study has confirmed previous observations that DCF and 2-CdA are equivalent in the treatment of HCL. The effectiveness of FA in the treatment of HCL is not as good as 2-CdA or DCF and only minor clinical experience has been reported so far [130]. 

### 3.2. Chronic Lymphocytic Leukemia

Fludarabine and 2-CdA are highly active in CLL, both previously treated and untreated [131,132,133,134,135,136,137,138,139,140]. The results of the studies presented so far have shown that both drugs give a similar CR rate and overall response (OR) rate, but the influence of these agents on survival times is still uncertain [136,138]. The CR rate induced with PNA is significantly higher than in patients treated with conventional chemotherapy. In refractory or relapsed patients FA has an OR rate of 12-73%, including CR in the range of 0-25% [136,137,138].

Cladribine has also been found to be quite effective in the treatment of patients with CLL, even when they are resistant to other therapies. The activity of 2-CdA in patients with CLL resistant to conventional treatment was first reported in 1988 by Piro *et al*. [140]. The OR was obtained in 10 of 18 patients. In our study among 184 pretreated patients, a CR was obtained in 23 (12.5%) and a PR in 66 (35.9%), giving an OR rate of 48.4% (44). Pentostatin has been also less extensively tested in CLL than FA. In several series this agent demonstrated about 25% response rate [138].

The results of randomized phase III trials indicate that PNA are more active than alkylating agents in previously untreated CLL patients [131,132,133,134,135]. Rai *et al*. [136] have published the results of a long-term randomized, multicenter trial comparing FA with Chl in advanced CLL. They randomized 509 patients with Rai stage I and II CLL with disease-related symptoms and all patients in the high-risk category (Rai stage III or IV). Patients were randomly assigned to one of the three arms: FA, chlorambucil or FA plus chlorambucil. Assignment of patients to the FA plus Chl group was stopped after the interim analysis because of the excessive toxicity and a response rate not better than after FA alone. Among the other two groups the OR rate was significantly higher for FA (63%) than for Chl (37%) (p < 0.001). The CR was also higher in FA group (20%) than in the Chl group (4%) (p < 0.001). The median duration of response and median PFS of the patients treated with FA was 25 months and 20 months, respectively. In contrast both values were 14 months in the Chl group. However, there was no significant difference in the median overall survival in the both groups (66 and 56 months in FA and Chl group, respectively) (p = 0.21). 

High CR and OR rates in CLL patients treated with 2-CdA as first-line therapy were also obtained in a prospective, randomized, multicenter trial [132,133]. In this study we compared the efficacy and toxicity of 2-CdA with prednisone and Chl plus prednisone in previously untreated patients with progressive and advanced CLL. Of 229 evaluated patients, 126 received 2-CdA with prednisone and 103 received Chl with prednisone. The data obtained from this trial indicate that the OR rate after 2-CdA and prednisone therapy was significantly higher than that after Chl and prednisone treatment (97% and 57%, respectively, p < 0.001). Moreover, the clinical OR rate after 2-CdA was also significantly higher (47%) than that after Chl treatment (12%) (p < 0.001). Likewise, progression-free survival (PFS) was significantly longer in the 2-CdA treated group (p = 0.01). However, there was no difference in overall survival duration between the two groups and no difference in event-free survival. 

The treatment of outpatients is more convenient when oral formulation of chemotherapeutic agents is available. Recently, an oral formulation of FA was developed with equivalent efficacy and tolerability to the i.v. formulation [142]. The pharmacokinetic studies demonstrated that FA at the oral dose of 40 mg/m^2^ used a once-daily provides a systemic exposure similar to that of FA administered i.v. at the dose of 25 mg/m^2^/day 

Among cytotoxic agents alkylating drugs and anthracyclines were the most frequently combined with PNA. O’Brian *et al*. [143] have treated a total of 128 CLL patients with FA at a dose of 30 mg/m^2^ for 3 days and CY at either 500, 350 or 300 mg/m^2^/day (FC program for three days). Some of these patients were previously untreated; others had been treated with alkylating agents and/or FA. More than 80% of patients not refractory to FA alone responded to combined treatment and a 38% response rate was observed in patients who were refractory to FA given earlier as monotherapy. In previously untreated patients, a CR rate (35%) was similar to that observed earier in CLL patients treated with FA alone. However, in previously untreated patients treated with FA and CY who obtained a CR, minimal residual disease (MRD) was seen in only 8% and the median time to progression had not been reached after the median observation of 41 months. Higher efficacy of the FC protocol compared with FA alone has been confirmed in three phase III trials of treatment naive patients with advanced/progressive CLL. [144,145,146,147]. The German CLL Study Group have compared FC regimen with the standard regimen of FA monotherapy in first line treatment of younger than 66 years patients with advanced CLL [145]. In total, 375 patients were randomized to the arm treated with FA (25 mg/m^2^/day for five days) or to the arm treated with FA (30 mg/m^2^/day) plus CY (250 mg/m^2^/day) on days one to three every 28 days. Of 207 patients evaluated for response, OR was higher in the FC regimen (94%) than in the FA group (83%; p < 0.01), and CR was 24% versus 7%, respectively (p = 0.01). The median PFS was 20 months in patients treated with FA alone and 48 months in patients treated with the FC regimen (p = 0.001). Thus far, no difference in median overall survival (OS) has been observed. Similar results were obtained in the US Intergroup Trial E2997 [145]. Symptomatic previously untreated patients with CLL were treated with FA alone or FA plus CY. A total of 278 patients were randomly assigned in this intergroup study. FC combination chemotherapy results in a significantly higher CR rate (23.4%) and an OR rate (24.3%) compared with FA alone (46% and 59.5%) (p < 0.01 and p = 0.013). FC treatment also resulted in longer PFS than those treated with FA alone (31.6 vs 19.2 months, p < 0.0001). However, FC regimen caused additional hematologic toxicity including more severe thrombocytopenia (p = 0.046) but did not increase the number of severe infections. In the UK LRF CLL4 trial, 777 previously untreated patients with CLL were randomly assigned to Chl, FA or to FC [147]. OR and CR rates were better with FC (94% and 38%, respectively) than with FA alone (80% and 15%, respectively), wchich were in turn better than with Chl (OR – 72%, CR – 7%). The PFS at five years was significantly better with FA + CY (36%) than with FA (10%) or Chl (10%; p < 0.00005). However, there was no significant difference in OS between patients given FC, FA alone or Chl. Moreover, patients had more neutropenia and days in hospital with FC or FA, than with Chll. Interestingly, there were less autoimmune hemolytic anemias with FC (5%) than with FA (11%) or Chl (12%). The results of these randomized trials indicate, that FC should now become the standard treatment for CLL and the basis for new protocols that incorporate monoclonal antibodies [147]. 

Cladribine combined with CY has been also investigated in phase II studies both in previously treated and untreated patients [148,149]. Montillo *et al*. [148] treated 29 patients with refractory or recurrent CLL or prolymphocytic leukemia (PLL) with 2-CdA (4 mg/m^2^/day) for three days every four weeks. Eleven patients (38%) had a response with the median response duration being 12 months. We determined the effectiveness and toxicity of 2-CdA in combination with CY (CC regimen) in patients with previously untreated CLL [150]. In the analyzed group of 82 patients the OR rate was 87.8%, which included a CR rate of 29.3%. Minimal residual disease was detected in only six of 24 (25%) patients with CR. Grade 3-4 thrombocytopenia was seen in four patients (5%) and neutropenia in ten patients (12%). Severe infections were noted in 21 (25%) patients. Recently, we performed an interim analysis of a randomized study that compared the activity and toxicity of 2-CdA and CY (CC programe) versus FA and CY (FC program) in previously untreated progressive or symptomatic CLL [150]. The preliminary results of this study indicate that CC and FC programs used as a first-line therapy give similar CR (46.7%, vs 48.7%; p = 0.43) and OR (88.6% vs 85.0; p = 0.31) rates and both regimens have comparable toxicity.

Pentostatin combined with CY may be also more effective than when given as monotherapy. In one study refractory or relapsed CLL patients were treated with DCF (4 mg/m^2^) combined with CY (600 mg/m^2^ or 300 mg/m^2^). Both drugs were administered on day one of each cycle and cycles were repeated every three weeks for six courses [151]. There were 17 responses (74%), including four CRs. It is worth noting that the response rate was 77% in FA refractory patients. 

Recent clinical observations indicate that combinations of PNA with mitoxatrone (MIT), or MIT and CY, are also highly active regimens [152,153,154,155,156]. Tsimberidou *et al*. [152] have shown that addition of MIT to FA does not have a significant advantage over FA alone in previously treated and untreated patients with CLL. Bosch *et al*. [153] evaluated FA combined with CY and MIT (FCM) in 60 patients with resistant or relapsed CLL. Overall, 30 (50%) patients achieved a CR, including 10 (17%) cases of negative MRD. The PR was achieved in 10 (17%) patients. The median duration of response was 19 months. The FCM regimen was well tolerated and could be administered on an out-patient basis. The results obtained with the FCM regimen appear to be better than those reported with FA alone. 

In our study we determined the efficacy and toxicity of 2-CdA, MIT and CY combination (CMC protocol) in treated and untreated patients with CLL [154,155,156]. The OR rate was 37% in previously treated patients and 69% in untreated patients and a CR rate was 5% and 29%, respectively.

Recently, we performed a randomized multicenter study to compare the CMC with CC and 2-CdA alone in previously untreated CLL patients [156]. Compared with 2-CdA alone CMC induced higher CR rate (36% vs 21%; p = 0.004), and a trend for a higher CR rate with CC was observed (29% vs 21% p = 0.08). In addition, the percentage of patients who were in CR and were MRD negative was higher in CMC compared with 2-CdA (23% vs 14%, p = 0.042). However, there were no differences in OR, PFS and OS survival among treatment groups. 

Monoclonal antibodies and chemotherapy have synergistic activity in patients with CLL [157,158]. Several recent reports suggest that in patients with CLL, rituximab combined with PNA can increase the OR and CR and prolonge PFS as compared with PNA or rituximab alone with acceptable toxicity [158,159,160,161,162,163,164]. The addition of rituximab to a variety of chemotherapy regimens for the treatment of patients with CLL has yielded promising results in phase II and III trials. 

The combination of rituximab with FC (R-FC regimen) demonstrated particularly high rates of OR, CR, PFS, and overall survival in previously untreated and relapsed/refractory CLL [161,162]. In order to validate this concept the German CLL study group (GCLLSG) initiated a multicentre, multinational phase III trial, CLL8, to evaluate the efficacy and tolerability of R-FC versus FC for the first-line treatment of patients with advanced CLL [161]. 817 patients were randomly assigned to receive 6 courses of either FC of R-FC. At the time of analysis (June 2008) the median observation time was 25.5 months (mo). The overall response rate was significantly higher in the R-FC arm (95%; 370/390) compared to FC (88%; 328/371 (p = 0.001). The complete response rate of the R-FC arm was 52% as compared to 27.0% in the FC arm (p < 0.0001). The PFS was 76.6% at two years in the R-FC arm and 62.3% in the FC arm (p < 0.0001). There was a trend for an increased OS rate in the R-FC arm (91% vs 88% at two years p = 0.18). 

In REACH trial 552 relapsed or refractory patients from 17 countries were randomized (1:1) to receive either R-FC or FC [162]. A median of one prior treatment had been administered, consisting of single-agent alkylator therapy (66%), purine-analogs (16%), or combination treatments (CHOP, COP, F-containing, 18%). Patients with prior FC combination treatment or prior rituximab were not eligible. Median observation time was 25 months. The primary endpoint PFS was significantly prolonged by median 10 months in the R-FC arm (30.6 months) compared to FC (20.6months, p = 0.0002). Secondary endpoints showed similar results. The OR rate was higher for R-FC vs. FC (70% vs. 58%, p = 0.0034), due to superior CR rates (24% vs. 13%, p = 0.0007). Grade 3/4 adverse events were higher in the R-FC arm (80%) vs. FC (74%), but serious adverse events were similar (50% vs. 48%, respectively). Grade 3/4 neutropenia and febrile neutropenia were only marginally increased for R-FC (42% and 15%) vs. FC (40% and 12%, respectively). The same correlation was seen for thrombocytopenia (R-FC 11% vs. FC 9%). Grade 3/4 infections (R-FC 18%, FC 19%) were similar, and there was no difference in bacterial, viral, or fungal infections between the two arms. Grade 3/4 anemia was slightly increased in the FC arm (R-FC 2%, FC 5%). 

### 3.3. Prolymphocytic leukemia

Prolymphocytic leukemia (PLL) is characterized by splenomegaly and high circulating lymphoid cell counts, these being medium-sized B or T lymphocytes with prominent nuclei [165,166]. B-cell (B-PLL) and T-cell (T-PLL) have some clinical features and cell morphology in common. However, their biology and pathology are different and they are distinct entities [166]. Both B- and T-PLL are rare and account for approximately 2% of chronic lymphoid leukemias. Approximately 80% of the cases are of B-cell phenotype. PLL is a disease of the elderly, with median ages of about 65 - 70 years. In contrast to HCL, the clinical course of PLL is progressive in the majority of patients, due to the resistance of the disease to conventional chemotherapy [167]. T-PLL has a more aggressive course, poorer response to chemotherapy and shorter median survival than B-PLL. Similarly to other indolent lymphoid malignancies, treatment does not seem to be indicated in asymptomatic patients with slowly progressive lymphocytosis. However, therapy is recommended in patients with symptomatic and progressive disease. In previous decades, splenectomy, splenic irradiation, leucapheresis, and alkylating agents used alone or in combination with other cytotoxic agents, have been used for the treatment of PLL. Purine nucleoside analogs have subsequently been used in the treatment of this disorder [169,170,171,172,173]. Palomera *et al*. [168] described the previously untreated patients with T- cell PLL, who received three cycles of 2-CdA and obtained CR. DCF 4 mg/m^2^ weekly or every two weeks have been used to treat small number of patients with B- or T-PLL with promising results. PR has been achieved in about half of the patients. The response rate in previously treated and untreated patients was similar. Several other reports have documented response to PNA in individual patients with PLL but long term follow-up is required to further evaluate the efficacy of these agents in this leukemia [169,170,171,172,173].

### 3.4. Acute lymphoblastic leukemia

Clofarabine is the most promising PNA in current clinical trials in pediatric and adult patients with hematological malignancies. During phase I studies the antileukemic activity at tolerable doses was well established thus several phase II clinical trials with clofarabine alone or in combination with other cytotoxic drugs have been recently conducted [174,175,176]. 

Jeha *et al*. [81] conducted a first pediatric trial of clofarabine in relapsed or refractory ALL and AML patients. In total, 17 patients with ALL and eight with AML were entered into this study. Clofarabine’s doses ranged from 11.25 to 70mg/m^2^ i.v. daily for five days. Five patients (20%) achieved a CR, and 3 (12%) achieved a PR, for an OR rate of 32%. Based on the results of phase I clinical trials with clofarabine in pediatric patients with advanced leukamias, the MTD was established as 52 mg/m^2^ daily for five days. The DLT was reversible hepatotoxicity and skin rash at 70mg/m^2^ i.v. per day for five days [81]. 

A phase II multicenter studies were performed in children with refractory or relapsed ALL or AML. The OR rate was 31% in ALL patients including six CR, four CR_p_ (complete response with incomplete platelet recovery), and five PR. The median survival was 42 weeks for responding ALL patients.The most clofarabine- related adverse events were febrile neutropenia, diarrhea, nausea and vomiting, fever, skin rash, headache, elevation in liver enzymes and bilirubin, infusion-related flushing and anxiety [81].

In a phase II, multicenter study, 61 children with refractory or relapsed acute ALL received CAFdA at a dose of 52 mg/m^2^ i.v. over hours daily for five days, every two to six weeks [177]. The response rate was 30%, consisting of seven CR, five CR_p_, and six PR. The most common adverse events of grade 3-4 were febrile neutropenia, anorexia, hypotenstion, and nausea. Based on this study, clofarabine was approved by the US FDA in December 2004 for the treatment of pediatric patients with relapsed or refractory ALL after at least two prior chemotherapy regimens. 

Recently Gidwani *et al*. [178] have published the results of the first report of successful remission induction in multiple relapsed ALL in a 9-year-old boy after combined treatment with CAFdA and arabinoside cytosine (ara-C).

Clofarabine was also used in combination with alkylating agents based on the results of preliminary studies showing synergistic cytotoxicity and inhibition of DNA repair for combining regimen. Eighteen patients with relapsed acute leukemias were treated with CY alone at the dose of 200mg/m^2^ on day 0, and with CAFdA at the dose of either 20 mg/m^2^ or 10 mg/m^2^ plus CY on day one [179]. Pharmacodynamic end points along with clinical results suggest usefulness of this combination strategy, whereas toxicity data suggest reduction of chemotherapeutic intensity

Nelarabine is a potent agent for treatment of hematologic malignancies with major efficacy in T-cell disorders [180,181]. Kurtzberg *et al*. [181] reported the clinical outcome of pediatric and adult patients with refractory hematological malignancies treated with nelarabine. The OR rate was 31%, however this rate was 54% in the subgroups of patients with T-cell ALL who achieved a CR or PR after one or two cycles of nelarabine. 

A phase II trial in 106 pediatric patients with relapsed or refractory T-cell malignancies revealed the effectiveness of nelarabine with an objective response rate of more than 50% in patients with first bone marrow relapse [182]. The initial nelarabine dose was 1.2 g/m^2^ daily for five consecutive days every 21 days. The most significant side effects of nelarabine were neurologic complications. 

In one pilot trial, a combination of nelarabine and FA was evaluated in 13 patients with a variety of refractory hematologic malignancies. Nine patients had indolent leukemias, including six with failed prior FA therapy, two patients had T-ALL, one had CML, and one had mycosis fungoides. Nelarabine was infused on days one, three and five at a dose of 1.2g/m^2^. On days three and five, FA at a dose of 30 mg/m^2^ was administered four hours before the nelarabine infusion. Seven patients (54%) responded to this combination regimen achieving a PR or CR. Additionally, among six patients who were refractory to PNA (2-CdA or FA), four had remission and one achieved stable disease. This result may suggest the usefulness of nelarabine in diseases refractory to other PNA. Nelarabine was accepted by European Medical Agency (EMA) in June 2005 and the FDA granted accelerated approval for this this drug in October 2005 for treatment of refractory patients with T-ALL/T-LBL or in relapsed patients after at least two prior regimens [180]. This use is based on the induction of a CR.

Recently, Furman *et al*. [183] have presented spectacular results of forodesine in a phase IIa, multicenter trial in patients with advanced precursor T-ALL or T-PLL. Forodesine was administered intravenously, at the dose of 40mg/m^2^ for five days weekly for total of six cycles. In nonresponders, after two cycles the dose was escalated to 90 mg/m^2^. At the last interim report, in total 34 pretreated patients an OR rate was 32.4% and a CR was achieved in 20.6%. Time to progression for CR patients was 77 to 398 days and an OS were 77 to 459 days. In the analyzed group only two patients died. Authors concluded, that forodesine used as a single agent in relapsed or refractory T-cell leukemia occurred effective with minimal toxicity. 

In the consecutive report forodesine was given at 40 mg/m^2^ for five days up to six cycles in three patients with refractory/relapsed T-ALL (two patients were prior and one post allogeneic hematopoietic stem cells transplantation) [184]. Up to publication of the study all three were alive and in CR with survival of 215+, 398+ and 180+ days, respectively. In authors opinion, forodesine used in monotherapy can be effective before and after allogeneic HSCT with minimal toxicity and without affecting potential graft versus leukemia effect .

Preclinical data also showed an activity of forodesine in B-cell ALL. Results of those studies led to a phase I/II study. Up to now, the results of only two trials are available [185,186]. In the former one dose-escalation study in 6 patients with B-ALL is presented [185]. As many as five of them experienced hematological benefit, defined as a of tumor burden decrease. One of the patients, treated with a high dose of forodesine achieved a CR and two patients PRs. In the second study, 12 patients with refractory/relapsed B-ALL were treated with forodesine at the dose of 80 mg/m^2^. Two of patients achieved CR and there were no patients with a PR. The authors emphasize, that forodesine was well-tolerated with preliminary evidence of activity as a single agent in B-ALL [1986].

### 3.5. Non-Hodgkin lymphoma

Non-Hodgkin lymphomas are a heterogenous group of entities with different clinical presentations and treatment outcomes depending on histopathological diagnosis. PNA showed remarkable activity in low grade non-Hodgkin lymphomas (LG-NHL) [187,188,189,190,191,192,193,194,195,196]. These agents have been found to be more effective in previously untreated LG-NHL than in patients refractory to or relapsing after conventional therapy. Recently, one phase II randomized study performed on previously treated LG-NHL patients showed that 2-CdA and FA give similar response rate and duration. [187]. In relapsed/refractory LG-NHL patients 2-CdA induced durable responses with OR rates ranging from 32 to 76% and CR rates between 10% and 38% [188,189,190,191,192]. 2-CdA was even more active in previously untreated patients [193,194]. The OR rate was achieved in 64-88% and a CR in 25-32%. 2-CdA was effective in combination with alkylating agents and MIT in the treatment of refractory or relapsed advanced stage of LG-NHL [195,196,197].

Lower doses of 2-CdA (5 mg/m^2^/week) have been also investigated both in monotherapy [205198] and in combination with MIT [199,200]. The results proved that in indolent lymphoid malignancies, the reduced dose of cladribine is highly active and possibly better tolerated than this drug administered in standard dose. 

FA is also an active agent in LG-NHL. In two large phase III clinical studies by Zinzani *et al*. [201] and Hagenbeek *et al*. [202] with FA used as a first line treatment in LG-NHL patients, the 84-68% of ORs and 38-47% of CRs have been demonstrated. Also in relapsed/refractory LG-NHL patients both FA and 2-CdA induced durable responses, with OR rates about 70% and high CR rates (48% ad 38%, respectively) [203].

A randomized phase III study of FA *versus* traditional CY, vincristine and prednisone (COP) regimen in patients with recurrent LG-NHL has been also reported [204]. No significant difference either in survival or in a response rate in FA (OR 64%, CR 9%) versus COP (OR 52%, CR 7%) arms was observed.

FM (FA- + MIT) regimen was highly effective in early stages of mucosa-associated lymphoid tissue (MALT) lymphoma, with projected 100% of a 5-year OS [205]. Good response to 2-CdA in MALT lymphoma patients was also observed, Jagger *et al*. [206] reported results of 2-CdA treatment in 25 previously untreated patients with 34% CR rate, including 100% CRs in patients with gastric and 13% with extra-gastric disease localization.

Robak *et al* [207] presented the updated results of a study evaluating the feasibility, efficacy and toxicity of the cladribine combined with MIT and CY (CMC regimen) in patients with refractory or relapsed NHL. Thirty six patients, 13 with mantle cell lymphoma (MCL), eight with diffuse large B-cell lymphoma (DLBCL), five with follicular lymphoma (FL), three with small lymphocytic lymphoma (SLL), and four with T-cell lymphoma were enrolled to the study. The CMC protocol consisted of 2-CdA at a dose of 0.12 mg/kg in a 2-hour infusion on days one through three, MIT 10 mg/m^2^ i.v. on day one and CY 650 mg/m^2^ i.v. on day one. The CMC courses were repeated at 4-week intervals. Thirty three patients were available for evaluation of response. Overall response rate was 58% (95%CI, 41-75%). The median failure-free survival (FFS) was five months (range, 2 - 17 months). The median OS for the entire group was nine months (range, 0.1 - 77 months). There was a significant difference in OS between responders and nonresponders after CMC therapy (log rank test, p = 0.015). When different disease status before CMC treatment was considered, a trend toward longer survival of recurrent patients was observed (log rank test, p = 0.08). The study showed that the CMC regimen is effective salvage therapy with acceptable toxicity in heavily pretreated patients with NHL including MCL and DLBCL. 

Cladribine has been reported to be active as the first-line therapy or in first relapse among patients with MCL achieving a response rate of 58% [201]. Rummel *et al*. [200] reported increased efficacy of the combination treatment with reduced-dose of 2-CdA and MIT in previously untreated or relapsed patients with MCL. They achieved a response rate of 100% with a 44% of CR rate. High response rates to the combined chemotherapy in MCL patients confirm its superiority to monotherapy. 

Another purine analog, fludarabine, has been also shown to be effective in the treatment of low-grade lymphomas, and in particular of MCL [208,209,210]. Similarly to 2-CdA, FA seems to be more active if it is combined with CY or CY and MIT. The German Low-Grade Lymphoma Study Group obtained an OR of 46 % in relapsed or refractory MCL patients receiving the combination of FA, CY and MIT [215]. Recent studies indicate that the addition of monoclonal antibody, rituximab, increases the clinical effectiveness of standard chemotherapy [205,206,207,208]. Forstpointner at al*.* [208] achieved a higher overall response rate (79% vs 58%), a higher complete remission rate (33% vs 13%), and a superior OS at two years (90% vs 70%) in the relapsed/refractory LG-NHL patients treated with rituximab combined with chemotherapy (R-FMC) compared to those received the chemotherapy alone (FMC) . We have evaluated the feasibility, efficacy, and toxicity of combined regimens that consisted of either rituximab plus cladribine (2-CdA) (the RC regimen) or RC plus CY (the RCC regimen) in the treatment of patients with heavily pretreated, indolent lymphoid malignancies [211]. Fifty-four adult patients with recurrent or refractory, low-grade NHL were treated according to the RC/RCC regimens. Thirty-three patients with CLL, 12 patients with LG-NHL and nine patients with MCL entered the study. Thirty-three patients (61%) had recurrent disease after prior therapy, and 21 patients (39%) had refractory disease. Thirty-one patients were treated on the RC regimen, and 23 patients were treated on the RCC regimen. Six patients (11%) achieved a CR, and 33 patients (60%) achieved a PR. The RC and RCC regimens were highly effective and well tolerated modalities of treatment in heavily pretreated patients with indolent lymphoproliferative disorders. 

## 4. Side Effects and Tolerability

The tolerability profile of PNA is distinguishable from that of other cytotoxic agents. However, bone marrow suppression with prolonged thrombocytopenia, neutropenia and anemia is a common complication of the PNA. Moreover, treatment with PNA leads to a decrease in the CD4+/CD8+ ratio for an extensive period of time exceeding even 24 months [212]. In consequence, infections, including opportunistic ones, are frequent events and infections with fatal outcome have been reported. Infections are the most frequent side effect in patients treated with PNA [212,213,214]. The most common infection complications arising from PNA toxicity are respiratory tract infections with bacterial pathogens and unexplained fever [213,214,215,216,217,218,219,220,221,222,223,224,225,226]. However, several studies revealed the emergence of new pathogens typically associated with T-cell dysfunction including *Pneumocystis carinii*, *Listeria monocytogenes*, *Cytomegalovirus*, *Herpes simplex* and *Varicella zoster* virus, as well as *Mycobacterium tuberculosis* [214,215,216,217,218,219]. T-cell depletion and neutropenia are well-known factors predisposing to infection. They may occur as a consequence of extensive leukemic bone marrow involvement and are worsened by PNA therapy.

Reduction in myelosuppression and granulocytopenia may be achieved with the prophylactic use of growth factors following the PNA cycles. O’Brien *et al*. [220] showed that prophylactic administration of granulocyte–colony stimulating factor (G-CSF) to patients treated with FA may reduce the incidence of pneumonia, but does not interfere with the infections. G-CSF prophylaxis ameliorated neutropenia, allowed delivery of therapy on time and reduced the incidence of pneumonia especially in high-risk patients treated with FA [220]. It should also be remembered that prophylactic administration of hemopoietic growth factors may greatly increase the cost of treatment. In our department G-CSF is applied only in patients with CLL treated with PNA when neutropenia is lower than 1x10^9^/L and severe infection develops.

As an alternative some authors are in favor of prophylaxis with antibacterial and antiviral agents in patients treated with PNA alone and especially in combination with other agents [221,222]. This approach is intended to reduce the incidence of opportunistic infections. Therefore, some investigators recommend routine prophylaxis with trimethoprim/sulfamethoxole or pentamidine and acyclovir or valacyclovir, which can account for the lack of serious infections caused by *Herpes simplex* and *Varicella zoster* viruses, *Pneumocystis carini and Listeria monocytogenes* [223].

Some reports suggest that PNA may induce autoimmune hemolytic anemia (AIHA), especially in patients with CLL despite the reduction in leukemic clone [224]. However, in our randomized study the difference in frequence of AIHA in patients treated with 2-CdA or Chl was not statistically significant [132,133]. Leporrier *et al*. [135] reported true AIHA only in 3% of the patients treated with CHOP (cyclophosphamide, hydroxyrubicin, vincristine, prednisone), 1.5% treated with CAP (cyclophosphamide, adriamycin, prednisone) and 1.5% treated with FA. Thus, the results of prospective multicentre randomized studies do not support the conclusion that the risk of AIHA is higher in the CLL patients treated with PNAs than in patients treated with Chl or other alkylating agents based regimens. 

Prolonged immunosuppression related to the PNA treatment may increase the risk of secondary malignancies. Some authors observed secondary MDS/AML and other cancers in patients treated with these agents [225,226,227,228,229]. However, a retrospective analysis performed by Cheson *et al*. [228] in which they compared secondary tumours in CLL patients treated with FA has shown that this agent does not increase the risk of secondary neoplasms. In our randomized study comparing 2-CdA with Chl in previously untreated patients with CLL we observed secondary malignancies only in two patients treated with 2-CdA and in one patient treated with Chl. 

Recently, we have analyzed whether the treatment with 2-CdA during the course of CLL had an impact on the subsequent occurrence of either secondary solid tumors or Richer’s syndrome (RS) [229]. There were 1,487 eligible patients, 251 treated only with 2-CdA, 913 treated only with alkylating agents (AA) based regimens and 323 treated with both 2-CdA and AA. Fifty eight patients with secondary cancer or Richter’s syndrome were observed. The differences between 2-CdA, AA and 2-CdA+AA treated group were not significant (P > 0.05). Only lung cancer occurred significantly more frequently in the 2-CdA (4%) treated group as compared to only one patient (1.9%) treated with AA (p = 0.001 and p < 0.05 respectively). On the basis of these observations 2-CdA does not seem to increase the risk of secondary malignancies except in case of the lung cancer. 

## 5. Conclusions

The purine nucleoside analogues, cladribine, fludarabine and pentostatin, share similar structure and mechanism of cytotoxic action, such as induction of apoptosis. However, they exhibit also significant differences especially in their interactions with enzymes involved in adenosine and deoxyadenosine metabolism activity. The main mechanism of PNA cytotoxicity is apoptosis which can be induced and mediated by indirect DNA damage, activation of caspases or by triggering of caspase-independent pathway. Several *in vitro and in vivo* studies have shown a synergy between PNAs and other chemotherapeutic agents and monoclonal antibodies active in hematological malignancies. PNAs alone or in combinations with other agents are the drugs of choice in HCL, CLL and NHL. More recently, a novel group of PNAs has been developed. These agents include CAFdA, nelarabine and forodesine and they are currently under investigation in pre-clinical and clinical studies. Their active forms, metabolic properties and mechanism of action of nelarabine and forodesine are different from other PNA. All these three drugs have shown promising activity in patients with relapsed and refractory ALL. Clofarabine was approved for the treatment of relapsed or refractory ALL in the third line of treatment. Nelarabine is recommended for T-ALL and T-cell lymphoblastic lymphoma (T-LBL). However, the use of this drug is limited by potentially severe neurotoxicity. Forodesine is a new drug with mechanism of action different than the other PNA and it has shown activity in relapsed and refractory T- and B-cells leukemias and CTCL. Great hopes are currently set on the use of these drugs in the treatment of lymphoid malignancies in adult and in pediatric patients. 

## List of Abbreviations

2-CdA-	2-chloro-2’-deoxyadenosine; 2-chloro-9-(2’-deoxy-β-d-ribofuranosyl)-adenine; cladribine
5’-NT-	5’-nucleotidase
AA-	aplastic anemia
ADA-	adenine deaminase
Ado-	adenosine
AIF-	apoptosis-inducing factor
AIHA-	autoimmune hemolytic anemia
ALL-	acute lymphoblastic leukemia
AML-	acute myeloid leukemia
APAF-1-	apoptotic protease activating factor
Ara-C–	9-β-d-arabinofuranosylcytosine; cytarabine
Ara-G-	9-β-d-arabinofuranosylguanine; nelarabine
AUC-	area under the concentration-time curve
BCX-1777-	immucillin H; forodesine
CAFdA-	2-chloro-2’-fluoro-2’-deoxyadenosine; 2-chloro-9-(2’-deoxy-2’-fluoro-β-d-arabinofuranosyl)-adenine; clofarabine
Chl-	chlorambucil
CLL-	chronic lymphocytic leukemia
C_max_-	maximum plasma concentration
CML-BP-	chronic myelogenous leukemia blast phase
CR-	complete response
CTCL-	cutaneous T-cell lymphoma
CY-	cyclophosphamide
dAdo-	deoxyadenosine
DCF-	2’-deoxyocoformycin; pentostatin
dCK-	deoxycytidine kinase
dGK-	deoxyguanine kinase
dGuo-	2’-deoxyguanosine
DLBCL-	diffusse large B-cell lymphoma
DLT-	dose limiting toxicity
DNMT1-DNA	methylotransferase
DNR-	daunorubicin
FA-	2-fluoroadenosine; 2-fluoro-9-(β-d-arabinofuranosyl)-adenine; fludarabine
FAMP-	fludarabine monophosphate
FC-	fludarabine + cyclophosphamide
FCM-	fludarabine + cyclophosphamide + mitoxantrone
FL-	follicular lymphoma
FM-	fludarabine + mitoxantrone
FMC-	fludarabine + mitoxantrone + cyclophosphamide
G-CSF-	granulocyte colony stimulating factor
HCL-	hairy cell leukaemia
hCNT-	human concentrative nucleoside transporter
hENT-	human equilibrative nucleoside transporter
LG-NHL-	low grade non-Hodgkin’s lymphoma
MALT-	mucosa associated lymphoid tissue lymphoma
MCL-	mantle cell lymphoma
MRD-	minimal residual disease
MDS-	myelodysplastic syndrome
MIT-	mitoxantrone
MTD-	maximum tolerated dose
NHL-	non-Hodgkin’s lymphoma
OR–	overall response
OS-	overall survival
PARP-	poly ADP-ribose polymerase
PFS-	progression free survival
PLL-	prolymphocytic leukemia
PNA-	purine nucleoside analogues
PNP-	purine nucleoside phosphorylase
PR-	partial response
RCC-	rituximab + cladribine + cyclophosphamide
R-FC-	rituximab + fludarabine + cyclophosphamide
RR-	ribonucleotide reductase
SLL-	small lymphocytic lymphoma
T_1/2_-	half life time
V_d_-	value of distribution
WM-	Waldenström macroglobulinemia
